# Speciation of Inorganic Compounds in Aquatic Systems Using Diffusive Gradients in Thin-Films: A Review

**DOI:** 10.3389/fchem.2021.624511

**Published:** 2021-04-06

**Authors:** Josep Galceran, Yue Gao, Jaume Puy, Martine Leermakers, Carlos Rey-Castro, Chunyang Zhou, Willy Baeyens

**Affiliations:** ^1^Departament de Química, Universitat de Lleida and AGROTECNIO-CERCA, Lleida, Spain; ^2^Analytical, Environmental and Geo-Chemistry Department, Vrije Universiteit Brussel, Brussels, Belgium

**Keywords:** trace metal speciation, DGT, diffusive domain, kinetic model, mobility, lability

## Abstract

The speciation of trace metals in an aquatic system involves the determination of free ions, complexes (labile and non-labile), colloids, and the total dissolved concentration. In this paper, we review the integrated assessment of free ions and labile metal complexes using Diffusive Gradients in Thin-films (DGT), a dynamic speciation technique. The device consists of a diffusive hydrogel layer made of polyacrylamide, backed by a layer of resin (usually Chelex-100) for all trace metals except for Hg. The best results for Hg speciation are obtained with agarose as hydrogel and a thiol-based resin. The diffusive domain controls the diffusion flux of the metal ions and complexes to the resin, which strongly binds all free ions. By using DGT devices with different thicknesses of the diffusive or resin gels and exploiting expressions derived from kinetic models, one can determine the labile concentrations, mobilities, and labilities of different species of an element in an aquatic system. This procedure has been applied to the determination of the organic pool of trace metals in freshwaters or to the characterization of organic and inorganic complexes in sea waters. The concentrations that are obtained represent time-weighted averages (TWA) over the deployment period.

**Graphical Abstract:**
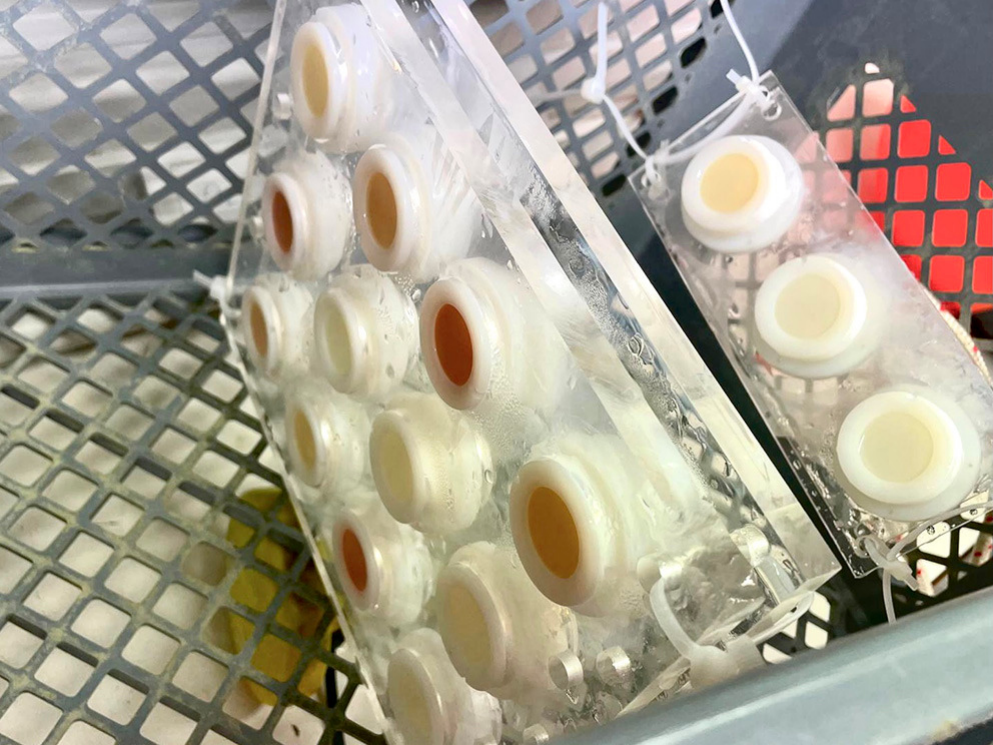


## Introduction

Trace elements occur in a variety of chemical forms in natural aquatic systems. The dynamics of these natural systems induce transfers from one chemical form into another one, so that the relative importance of each of these chemical forms changes with time. In the water column, phytoplankton blooms can produce high amounts of organic compounds that can bind to trace elements forming very strong complexes, but, in the sediments, diagenetic processes influence the distribution of the trace element species much more. The mineralization of organic matter and the corresponding reduction of a suite of electron acceptors control the exchange between the dissolved and solid phases.

The chemical forms which are most interesting to be studied in solution are the free ion, the kinetically labile (usually weakly bound) complexes, the non-labile (usually strongly bound) complexes, and the colloidal fraction. Several studies demonstrated that the free ion and some labile complexes are the most easily assimilated by organisms and, hence, they constitute the bioavailable fraction (Schintu et al., 2010; Sondergaard et al., 2014; Kim et al., 2016; Vannuci-Silva et al., 2017). In natural aquatic systems, the bioavailable fraction is also the toxic fraction, when the element is considered as toxic, or the micronutrient fraction, if the element is an essential element. We must notice that some elements, such as Cu or Cd, may change from micronutrient at low concentration to a toxicant at higher concentrations.

It is, thus, clear that the total dissolved element concentration cannot be used to inform us about the bioavailable amount and, thus, neither about its risk for the ecosystem, if it is a toxic element, nor about the limitation of phytoplankton growth, if it is an essential element. Speciation techniques to distinguish between the various chemical forms of an element are, thus, definitely required. There exists no analytical technique that can measure all chemical forms of an element in solution, hence it is necessary to combine several methods to obtain a general picture. The free ion concentration is, for some elements, especially in open ocean water, extremely low. There are some techniques designed to specifically measure free concentrations like ISE, AGNES, and DMT although there are some restrictions related to the cations that can be analyzed, to the salinity range, or to the limit of detection (Bakker et al., 2000; Temminghoff et al., 2000; Galceran et al., 2004; Pesavento et al., 2009; Weng et al., 2011; Chito et al., 2012; Companys et al., 2017). Concentrations of labile complexes can be assessed using another set of techniques. Some of them, like ASV or SSCP (van Leeuwen and Town, 2003; Town and van Leeuwen, 2019; Cindric et al., 2020), are of voltammetric nature, but there is an increasing use of techniques based on membranes. These techniques involve the dissociation of the labile complexes followed by the non-reversible trapping of the released ions in a solid substrate such as a resin. The amount bound to the resin is afterwards measured with a specific element analyzer such as ICP-MS. An example of a technique that can pre-concentrate labile metal or metalloid complexes is the Diffusive Gradients in Thin-films (DGT) technique (Zhang and Davison, 1995). The labile fraction depends on the particular instrumental characteristics (van Leeuwen et al., 2005), since each technique defines an operational time window for the dissociation. Complexes that fit into this window are labile in this technique, but could be less labile in another one defining a shorter time scale. The direct determination of non-labile or strongly bound element complexes and colloids is more difficult. Since colloids have a size between 1 nm and 1 μm (compounds with a molecular weight of 300–500,000 Dalton), by using ultrafiltration membranes of appropriate pore sizes, one can distinguish between colloids of various dimensions (Waeles et al., 2008), nevertheless, such measurements are mostly very laborious. The concentrations of the colloidal fraction(s) can, then, be measured with a specific element analyzer, after mineralization of the colloidal particles.

Strongly bound element complexes, which are mainly complexes involving organic ligands, can be destroyed by intense UV-light in acidic conditions.

In freshwater, the total dissolved concentration can be directly measured with minor or no sample treatment, if a technique such as ICP-MS is used, but this is not possible when marine samples are involved. For marine samples, often a SeaFast pre-concentration technique (Wuttig et al., 2019) is applied to eliminate the salt matrix and to increase the low concentrations often encountered in seawater samples.

The direct measurement of natural, uncontaminated water samples is often not feasible for trace metals, which are typically present in very low concentrations close to the femtomolar (fmol L^−1^, pg L^−1^) range. An alternative is to use *ex-situ* pre-concentration techniques like Solid Phase Extraction (SPE) with ion-exchange resins (Pohl, 2006), which can even be coupled to fractionation schemes such as (ultra)filtration, in order to obtain information about different speciation pools. However, the use of *in-situ* pre-concentrating passive samplers like DGT represents an advantage in terms of minimization of contamination and perturbation of speciation due to sample transport and management in the lab.

In this paper, we will mainly focus on the bioavailable fraction of Cd, Co, Cu, Fe, Hg, Mn, Ni, Pb, and Zn in the water column of aquatic systems. This means that the concentration of the free ion plus that of the labile complexes will be addressed. A tool that responds to that requirement is the DGT technique, which consists of a hydrogel diffusive layer backed by a resin layer. The composition of the hydrogel and the resin will be adapted to the kind of chemical compound to be determined. The sampling of labile complexes (and sometimes free ions) of the different metals with DGT will be discussed in detail. In addition, different models that can provide information (about the meaning of *c*_DGT_ and about the lability and dissociation constants of element complexes) are presented.

## Diffusive Gradient in Thin-films Technique

**Figure 1:**
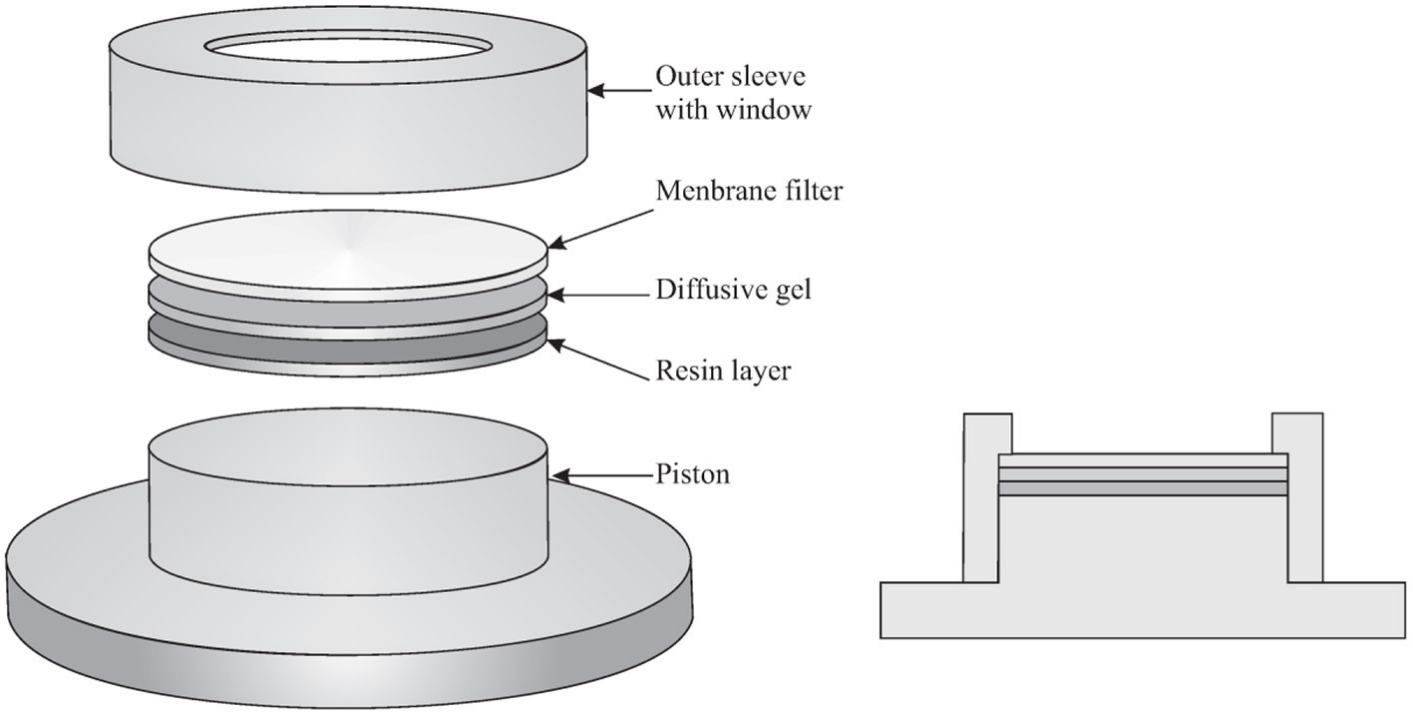
Schematic drawing of the DGT device (Adapted from Averós et al., 2020).

The DGT is a passive sampling technique and is preferred to other passive sampling techniques because its result is, in many instances, independent from the flow conditions in the aquatic system, in particular the turbulence. Indeed, the diffusive gel and filter in front of the binding layer define a region where diffusion is the only transport phenomenon. For more accurate determinations, a relatively small Diffusive Boundary Layer (DBL) in the sampled environment has to be added to the thicknesses of the diffusive gel and filter. A schematic drawing of the DGT is shown in Figure 1. The classic DGT device is composed of a cylindrical plastic molding (a cap and a piston base), holding together three successive layers, which are: a membrane filter, a diffusive hydrogel (polyacrylamide or APA hydrogel−0.8 mm thick), and a resin gel (binding Chelex®-100 binding resin−0.4 mm thick). The most common filters are 0.45 μm pore size cellulose acetate/HVLP Durapore−0.125 mm thick, but any filter membrane that is suitable for the element to be determined can be used. Hydrogels are used to guarantee a diffusive domain with a certain thickness where not only the analyte transport is diffusion controlled, but where also the dissociation of the labile complexes occurs. Hydrogels with several thicknesses can be used (0.2, 0.4, 0.8, 1.2 mm) and the thicker the diffusive domain, the longer the time that a complex has to dissociate. The characteristic time for an element M traveling through the diffusive domain equals:

(1) $$\begin{array}{l} t=\frac{{\left(\Delta g+\delta^{\text{dbl}}\right)}^{2}}{2D_{\text{M}}} \end{array}$$

where Δg is the aggregate thickness of diffusive gel plus filter (i.e., the thickness of the material diffusion layer), δ^dbl^ indicates the thickness of DBL, and *D*_M_ is the diffusion coefficient of the element (e.g., a metal) when assumed to be the same in solution as in the gel and filter. For well-mixed media, the DBL is small compared to the thickness of the diffusive gel and it can often be neglected.

For diffusive domain thicknesses of 0.5, 1.0, and 1.5 mm, a residence time in that domain of, respectively, 8, 33, and 75 min can be roughly estimated. Thus, the thicker the diffusive domain, the larger the labile fraction of metal species will be assessed. Hydrogels have distinct properties such as a high water content and controllable swelling behavior and are generally limited to two types for use in DGTs: polyacrylamide or agarose. They are synthesized via a polymerization process and, depending on the amount and type of cross-linker that is added, they will have smaller or larger pore-sizes (Zhang and Davison, 2000; Scally et al., 2006; Baeyens et al., 2011; Shiva et al., 2015; Turull et al., 2019). Polyacrylamide is the most commonly used gel, but for some elements that show interactions with this gel, it is replaced by agarose. A suite of resins can be used to bind the analytes (dos Anjos et al., 2017; Menegario et al., 2017).

A DGT device allows trace elements to diffuse through the diffusive gel and bind to the resin gel. Therefore, based on total amounts of accumulated elements on the resin gel during deployment, a labile solute concentration can be calculated using Fick's law, Equation (2), assuming a perfect sink condition (i.e., all ions arriving at the interface between the diffusive hydrogel and the resin gel are completely bound to the resin gel) and steady-state regime:

(2) $$\begin{array}{l} c_{\text{DGT}}=\frac{M\left(\Delta g+\delta^{\text{dbl}}\right)}{D_{\text{M}}At} \end{array}$$

where *c*_DGT_ is the DGT-labile metal concentration in the water, *M* is the mass of trace element accumulated on the resin gel, *A* is the exposure area of the DGT device, and *t* is the DGT deployment time.

Notice that Equation (2) is based on the first Fick's law assuming a fixed effective concentration in the solution. Thus, when only free metal is present in the solution, *c*_DGT_ stands for the total labile metal concentration in the solution and Equation (2) indicates that DGT allows this measurement with the knowledge of only one specific parameter of the analyte: the diffusion coefficient. When different metal species are present in the solution that can contribute to the transport and accumulation of the metal by dissociation, *c*_DGT_ stands for the apparent or effective free metal concentration in a solution that would give rise to the same accumulation than the sample (Galceran and Puy, 2015; Puy et al., 2016).

*c*_DGT_ can also be interpreted in terms of the real species present in the sample, as indicated in Equation (3) below. The lability degree, ξ_*j*_, quantifies the contribution of a given complex M^*j*^L to the actual flux received by the DGT in comparison with the maximum possible flux of this complex if it was fully labile (local equilibrium fulfilled all throughout the diffusion domain or, equivalently, infinitely large dissociation rate constant; Galceran et al., 2001; Puy and Galceran, 2017). ξ ranges between 0, for inert complexes, to 1, for fully labile complexes. If there are *h* parallel complexes (Galceran and Puy, 2015; Puy et al., 2016; Zhao et al., 2020).

(3) $$\begin{array}{l} c_{\text{DGT}}=c_{\text{M}}+\overset{h}{\underset{j=1}{\sum }}\xi_{j}\varepsilon_{j}c_{{\text{M}}^{j}L} \end{array}$$

where the normalized diffusion coefficients are

(4) $$\begin{array}{l} \varepsilon_{j}=\frac{D_{{\text{M}}^{j}L}}{D_{\text{M}}} \end{array}$$

By just taking all lability degrees at their maximum values (ξ_*j*_ = 1), Equation (3) reverts to the useful and widely-used concept of “maximum dynamic concentration” (Unsworth et al., 2006; Balistrieri and Blank, 2008; Bradac et al., 2009; Warnken et al., 2009; Davison and Zhang, 2012; Han et al., 2014; Zhang and Davison, 2015; Zhu and Gueguen, 2016; Macoustra et al., 2019; Cindric et al., 2020),

(5) $$\begin{array}{l} c_{\text{dyn}}^{\max}=c_{\text{M}}+\overset{h}{\underset{j=1}{\sum }}\varepsilon_{j}c_{{\text{M}}^{j}\text{L}}\geq c_{\text{DGT}} \end{array}$$

We turn now our attention toward the assessment and implications of the DBL. The velocity of a liquid flow becomes zero at the interface with a solid phase. This suggests to approximate the transport of the species in the vicinity of the solid surface as if there was just diffusion of the species in a stagnant region (DBL) extending from the solid surface up to a distance (δ^dbl^) where bulk concentrations are assumed to be restored by the flow and/or turbulences. With increasing rate of solution flow, the DBL layer becomes thinner, but it still exists even when the deployment solution for DGT devices is vigorously stirred. Therefore, to ensure optimal accuracy, DBL should be considered when calculating the concentration measured by DGT (Garmo et al., 2006; Warnken et al., 2006). It has been suggested that the thickness of the DBL can be assessed by using DGT devices with diffusive gels of different thickness. If DGT pistons equipped with different thickness of the diffusive gels, for example 0.40, 0.80, and 1.20 mm, are deployed in natural water systems, Equation (2) can be rewritten as:

(6) $$\begin{array}{l} \frac{1}{M}=\frac{\Delta g}{c_{\text{DGTe}}D_{\text{M}}^{\text{g}}At}+\frac{\delta^{\text{dbl}}}{c_{\text{DGTe}}D_{\text{M}}^{\text{w}}At} \end{array}$$

where the superscripts g and w refer to the diffusive gel (and filter) and water solution, respectively. The added subscript “e” stands for the extended definition of DGT given by Equation (6) [instead of Equation (2) where $D_{\text{M}}^{\text{g}}=D_{\text{M}}^{\text{w}}$ was assumed; Davison and Zhang, 2016]. Equation (6) suggests that plotting 1/*M vs*. Δ*g* enables the estimation of both *c*_DGTe_ and δ^dbl^, if the diffusion coefficients are known. If the behavior of 1/*M vs*. Δ*g* is actually linear, the slope (*s*) and the intercept (*b*) are given by:

(7) $$\begin{array}{l} s=\frac{1}{c_{\text{DGTe}}D_{\text{M}}^{\text{g}}At} \end{array}$$

(8) $$\begin{array}{l} b=\frac{\delta^{\text{dbl}}}{c_{\text{DGTe}}D_{\text{M}}^{\text{w}}At} \end{array}$$

However, a straight line assumes that *c*_DGTe_ is constant *vs*. Δ*g*, and this is not the case if the solution contains partially labile ML complexes, which dissociate more with increasing Δ*g*. Fortunately, the DBL is a physical quantity that depends on the flow pattern in the vicinity of the solid surface, but it is essentially independent of the nature and quantity of the analyte used in Equation (6). Therefore, in field conditions the DBL could be assessed with an analyte that forms no complexes, for example an organic compound such as E2 (17 beta-estradiol) (Guo et al., 2019) or a metal that forms only very labile complexes in the studied aquatic system (Warnken et al., 2007). This DBL is, thus, also valid for all other analytes that are assessed in the same aquatic system.

For a turbulent system, when the DBL is only about 10% of the diffusive layer thickness (Δ*g* is typically around 1 mm), the DBL can be considered negligible. In this case, Equation (2) can be simplified to:

(9) $$\begin{array}{l} c_{\text{DGT}}=\frac{M\Delta g}{D_{\text{M}}At} \end{array}$$

## Transition Metals

The elements most studied with the DGT technique are, without any doubt, transition metals such as Cd, Co, Cu, Fe, Mn, Ni, Pb, Zn, because they all have a good to strong affinity for the Chelex resin and polyacrylamide can be used as a suitable hydrogel (Zhang and Davison, 1995; Garmo et al., 2003; Scally et al., 2006; Gao et al., 2019). In some studies, mercury was also determined with Chelex resin and polyacrylamide but, in other studies, resins with thiol groups and agarose as the hydrogel were recommended (see the section Mercury Speciation). Therefore, mercury speciation will be separately discussed from the other transition metals. According to Bio-Rad Laboratories, the affinity order of the various metals for Chelex is Hg^+2^ >Cu^+2^ >>Pb^+2^ >Fe^+3^ >Ni^+2^ >Zn^+2^ >Co^+2^ >Cd^+2^ >Fe^+2^ >Mn^+2^, and the capacity of the resin is around 0.5 mmol/mL which means 15 μmol for a 0.4 mm thick standard resin gel. If the concentration of the trace metal in solution is 1 μmol/L, then after 1 day of DGT exposure with typical parameter values (*D* = 5 × 10^−6^ cm^2^/s; *A* = 3.14 cm^2^; diffusion domain thickness = 0.1 cm), the mass in the resin is 0.014 μmol. This means that just about 0.1% of the resin capacity is reached and that, assuming only this analyte present, it would take 1,000 days before capacity was reached.

(10) $$\begin{array}{l} \text{Log}D_{\text{T}}=1.37023\left(T-25\right)+8.36\times 10^{-4}{\left(T-25\right)}^{2}/\left(109+T\right)\\ +\text{log}\left(D_{25}\left(273+T\right)/298\right) \end{array}$$

There are three important factors that have an influence on the effective diffusion coefficients presented in Table 1, besides the temperature effect, which is expressed by Equation (10): the ionic strength of the solution, the pore size of the gel, and the size of the chemical compounds.

**Table 1:** Trace metal diffusion coefficients (in 10^−6^ cm^2^ s^−1^) in polyacrylamide gel (www.dgtresearch.com).

***T* (°C)**	**Cd**	**Co**	**Cu**	**Fe**	**Mn**	**Ni**	**Pb**	**Zn**
5	3.29	3.21	3.36	3.3	3.16	3.12	4.34	3.28
15	4.57	4.46	4.67	4.58	4.39	4.33	6.03	4.56
25	6.09	5.94	6.23	6.11	5.85	5.77	8.03	6.08

In addition to diffusion, at low ionic strengths (e.g., <1 mmol/L), some electrostatic effects can modify the transport of the analytes. At these conditions, the gel could have a small positive charge (leading to Donnan partitioning of cations at the gel surface) which depresses the metal concentration at the gel surface (Warnken et al., 2005). This lower surface concentration has the effect of lowering the net diffusion coefficient measured from solution to the resin, while the diffusion coefficient in the gel itself is actually unchanged. Since only a small percentage of world's freshwater systems have an ionic strength <1 mmol/L, measurements of trace metals—in poorly complexing media—by DGT should be rather straightforward to interpret. Besides the commented (occasional) electrostatic effects of the gel, the resin, especially in the case of Chelex, exhibits a high negative charge which induces a Donnan partitioning at the resin/diffusive gel interface (Puy et al., 2014). This partitioning will have an important effect on the accumulation of elements which have non-negligible concentrations of partially labile charged complexes, which will be pre-accumulated or excluded (depending on the sign of the electrical charge) from the resin domain (where complexes can penetrate, Mongin et al., 2011; Uribe et al., 2011). The electrostatic effects of the resin charge are also very relevant when perfect sink is not fulfilled for the target ion (Altier et al., 2016). When the metal concentration drops to zero at the resin/diffusive gel interface and complexes are labile, this effect can be neglected. This can be checked by using DGT devices with a stack of resin disks which are eluted separately.

Hydrogels with different pore sizes can be prepared by varying the concentrations and type of crosslinker. Scally et al. (2006) and Shiva et al. (2015) studied the diffusion coefficients of transition metal ions in classic open pore gel (0.12% cross-linker; pore size >5 nm) and restricted pore gel (0.8% cross-linker; pore size <1 nm). They found for the open pore gel *D*^g^/*D*^w^ ratios of about 85%, while for the restricted pore gel this ratio decreased to 60%. Thus, in restricted pore gel, the ion diffusion coefficient is about 70% of that in open pore gel. Since the radius of trace metal ions is smaller (<0.3 nm) than the restricted gel pore size (≈1 nm), the mechanism of retardation is likely due to the greater diffusional path length (tortuosity) within the restricted gel compared with the open pore gel (≈5 nm pore size).

Metal complexes are larger in size than metal ions, which can have an influence on their diffusion coefficients. Diffusion coefficient of Pb complexes decreased with increasing size of the ligand, in the order of diglycolic acid (DGA), nitrilotriacetic acid (NTA), fulvic acid (FA), and humic acid (HA) (Scally et al., 2006).

Diffusion coefficients of PbNTA and PbDGA showed a decrease of 20–30% in the open pore gel compared to that of the free metal, while this decrease is about 90% for PbHA. In freshwaters, fulvic species are generally more common and, for some metals, they may dominate the solution species. Provided they are sufficiently labile to dissociate while they traverse the diffusion layer and penetrate into the resin layer, which is usually the case, they will contribute to the mass of metal measured by open pore DGT with about 20% of the sensitivity of uncomplexed metal (Scally et al., 2006).

Several parameters have an influence on the Limits of Detection (LODs), such as the pre-conditioning/cleaning of all materials including filter, resin, and hydrogel, the quality of the chemical reagents (including the purity of water used for dilutions), the air quality in the laboratory and the measuring conditions (type of analysis instrument, stability of the instrument, background signal). Moreover, DGT-LODs are only indicative, because the longer the DGT is deployed in the aquatic system, the lower the LOD becomes. Nevertheless, it is useful to have an idea about the concentration levels of labile trace metals that can be measured for a fixed time period of deployment. In Table 2 we compare the blank value results of unexposed Chelex resin observed by Garmo et al. (2003) with those determined by Zhou and co-workers (Gaulier et al., 2021). The blank values are similar in both studies, except for Mn, Pb, and Ni while the STDs and LODs are similar, except for Co, Pb, and Fe. The largest variation, which is more than one order of magnitude, is observed for the LODs of Co and results from the large difference in standard deviation measured in the two cases.

**Table 2:** DGT blanks (with standard deviation STD) and Limits of Detection (LOD) for 24 h exposure time.

	**Reference 1**			**References 2 and 3**		
	**Mean blanks**	**STD**	**LOD DGT**	**Mean blanks**	**STD**	**LOD DGT**
	**ng**	**ng**	**ng/ml**	**ng**	**ng**	**ng/ml**
Cd	0.053	0.015	0.0032	0.059	0.017	0.0029
Co	0.100	0.19	0.037	0.036	0.0057	0.001
Cu	0.970	0.26	0.047	0.55	0.12	0.02
Mn	0.360	0.11	0.021	1.54	0.091	0.016
Ni	4.10	0.48	0.087	0.68	0.32	0.057
Pb	1.000	0.16	0.019	0.086	0.0076	0.001
Zn	6.30	2.2	0.58	8.55	1.58	0.27
Fe	17.5	5.6	2.6	5.51	0.72	0.12

*Reference 1 (Garmo et al., 2003); references 2 and 3 (Zhou, pers. Comm.) and (Gaulier et al., 2021). $LOD\text{ DGT}=\frac{3\sigma_{blank}\Delta g}{D_{\text{M}}^{g}At}$ where σ is the STD of the blank; t = 24 h; A = 3.14 cm^2^; Δg = 0.08 cm, and $D_{\text{M}}^{g}$ the diffusion coefficient*.

Numerous surveys of labile trace metals have been successfully carried out in fresh- and sea-water environments, but we will present here one case study of trace metal speciation in each of those aquatic systems.

### Speciation Results in Marine Water Ecosystems

**Figure 2:**
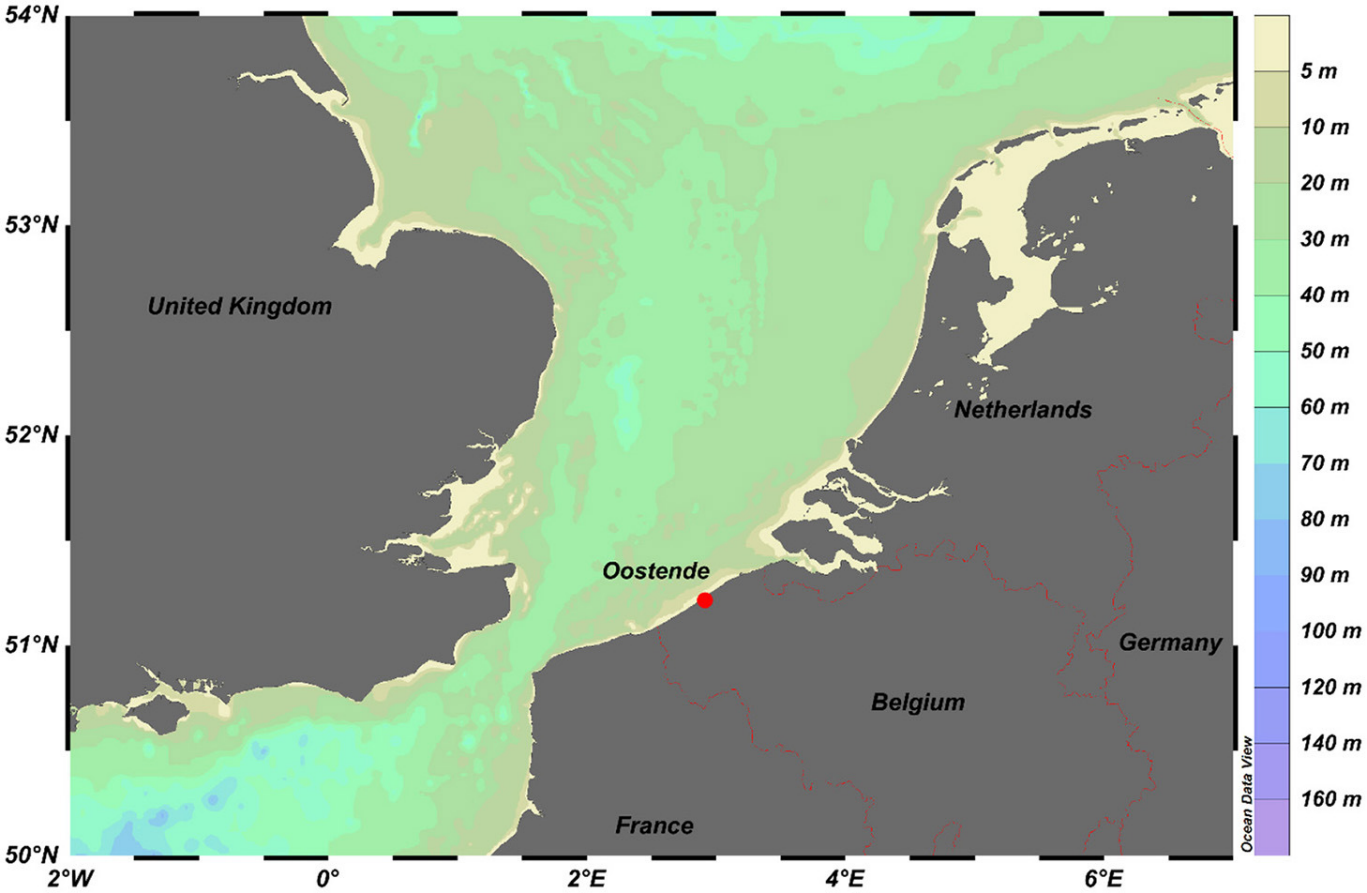
Sampling station Oostende at the Belgium coast (Schlitzer, Reiner; Ocean Data View, https://odv.awi.de, 2020).

In coastal areas, not only metal levels are generally higher than in offshore areas, but also other abiotic and biotic parameters are different, which can have an influence on the metal speciation. Therefore, the Oostende sampling site (see Figure 2), which is a coastal station at the Belgium coast that is heavily influenced by anthropogenic inputs, was selected for assessing the metal fraction that is labile (and, hence, also more bioavailable) and for comparing these labile metal fractions to those found in an offshore area in the Mediterranean Sea (Gao et al., 2019). Diffusive Gradients in Thin-films devices with different thicknesses of the diffusion domain allow one to provide information on the labile metal fractions at the coastal station and in the offshore area. The ratios of the field to blank concentrations in the Belgian Coastal Zone (BCZ) (Gaulier et al., 2021) are higher than in the Mediterranean Sea except for Cd (Baeyens et al., 2018). All ratios are higher than 8, except for Cd in the BCZ and Fe in the Mediterranean Sea, which are high enough to calculate accurate concentrations. The DGT concentrations of Cd and Fe in seawater can be very low, while, on the other hand, the blank values of the commercial Chelex resin in DGT can be high, resulting in those low ratios. However, when an acid pre-treatment of the resin is performed, all blank concentrations decreased and, for example, the field-to-blank ratio of Fe in the Mediterranean Sea became also higher than 10. During the same Mediterranean Sea cruise, the reproducibility of the DGT samplers was also investigated. For the classic (*n* = 5; hydrogel layer thickness is 0.8 mm) as well as the fast DGT (*n* = 2; no hydrogel layer) the relative standard deviations (RSD) for Cd, and Cu were below 20%. For Fe, that only could be determined with the fast DGT, the RSD was 33%.

**Figure 3:**
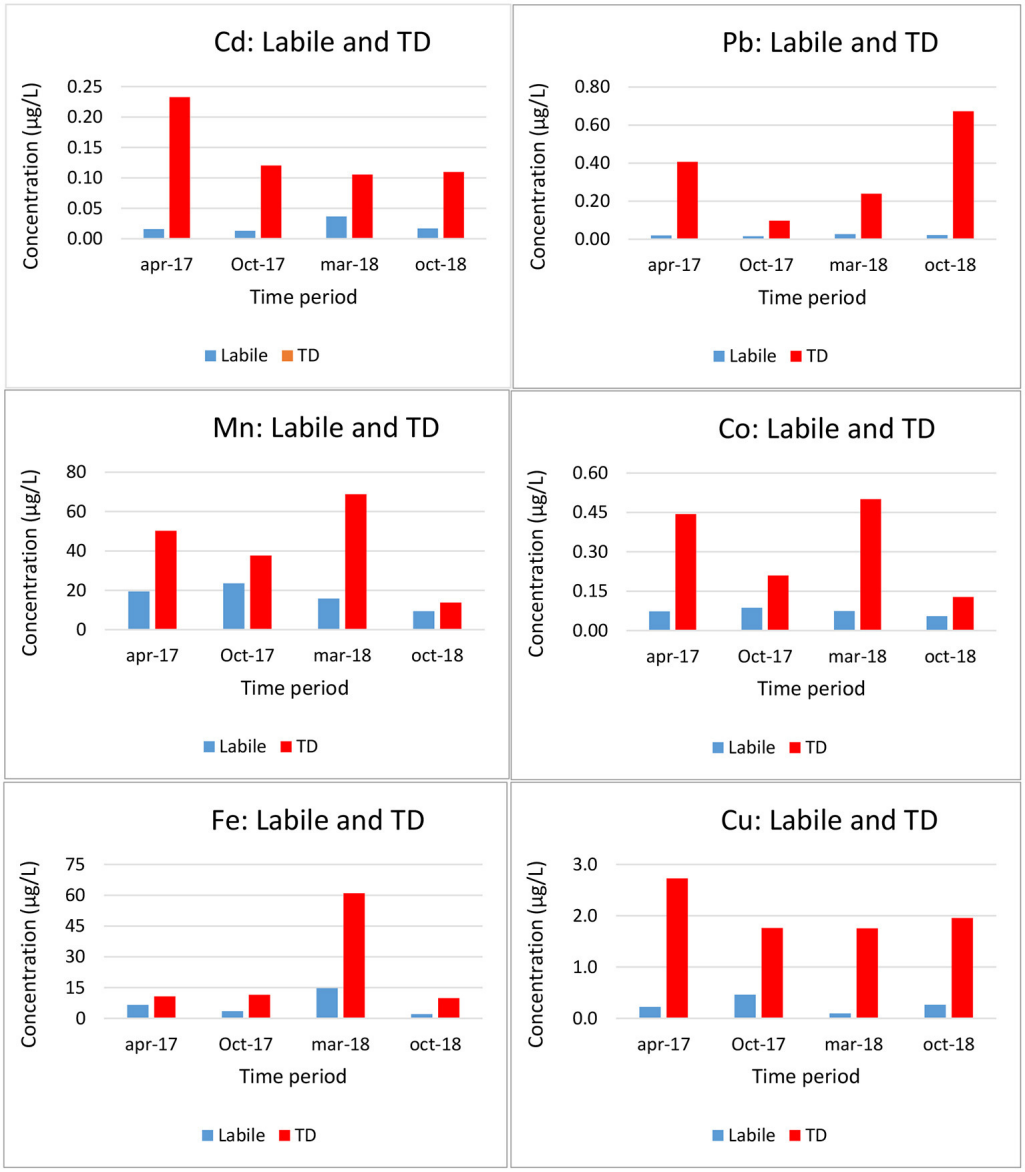
Total dissolved (TD) and labile (DGT) trace metal concentrations at station Oostende in the Belgian Coastal Zone (Gao et al., 2019).

The DBL was taken as 0.2 mm (Guo et al., 2019). Total dissolved (TD) and labile metal (classic DGT, with a Δ*g* = 0.8 + 0.125 mm) concentrations at the coastal station of Oostende are shown in Figure 3. As expected, labile concentrations are lower than TD concentrations. The labile concentrations (relative to the TD) are low for Pb and Cu, and high for Mn, as expected.

Two metal-complex pools are considered: one, labeled inorganic ML_in_, for smaller molecules and another one, labeled organic ML_org_. The normalized diffusion coefficients of these pools, are ε_in_ and ε_org_. The total dissolved amount can be considered as the summation of the free metal pool *c*_M_, the inorganic pool *c*_ML,in_, and the organic pool *c*_ML,org_.

(11) $$\begin{array}{l} c_{\text{T},\text{M}}=c_{\text{M}}+c_{\text{ML,in}}+c_{\text{ML,org}} \end{array}$$

Total dissolved metal concentrations (*c*_T,M_) and percentages of free metal, inorganic complexes and organic complexes were taken from the literature (see Table 3). Conditional stability constants (in excess of ligand) can be used, e.g., for the inorganic pool

(12) $$\begin{array}{l} K_{\text{in}}^{\prime }=\frac{c_{\text{ML},\text{in}}}{c_{\text{M}}} \end{array}$$

Equation (3) can be invoked for this particular case, i.e., *c*_DGT_ is split as a summation of the labile fractions, each weighted by its normalized diffusion coefficient ε_*j*_. For the different configurations used in these experiments (no hydrogel, 0.4 and 0.8 mm thick diffusive hydrogel layer) DGTs, Equation (3) becomes:

(13) $$\begin{array}{l} c_{\text{DGT}}^{\text{g}0}=c_{\text{M}}+\varepsilon_{\text{in}}\xi_{\text{in}}^{\text{g}0}c_{\text{ML,in}}+\varepsilon_{\text{org}}\xi_{\text{org}}^{\text{g}0}\;c_{\text{ML,org}} \end{array}$$

(14) $$\begin{array}{l} c_{\text{DGT}}^{\text{g}4}=c_{\text{M}}+\varepsilon_{\text{in}}\xi_{\text{in}}^{\text{g}4}c_{\text{ML,in}}+\varepsilon_{\text{org}}\xi_{\text{org}}^{\text{g}4}\;c_{\text{ML,org}} \end{array}$$

(15) $$\begin{array}{l} c_{\text{DGT}}^{\text{g}8}=c_{\text{M}}+\varepsilon_{\text{in}}\xi_{\text{in}}^{\text{g}8}c_{\text{ML,in}}+\varepsilon_{\text{org}}\xi_{\text{org}}^{\text{g}8}\;c_{\text{ML,org}} \end{array}$$

where ξ_*j*_ is the lability degree for each metal complex pool (*j* = in or org) for the different DGT configurations (superscripts g8, g4, or g0, see Table 3). Neglecting mixture effects (Altier et al., 2018), one can approximate the true ξ in a mixture of ligands (or pools, in this case) with the ξ computed for the case where there is only one ligand (i.e., one pool). Assuming excess of ligand and perfect-sink conditions, the lability degree can be written as a function of the dissociation rate constant (*k*_d_), the normalized diffusion coefficient of the complex and the thicknesses of the just-diffusion (gel disc and filter, Δ*g* + δ^dbl^), and of the reaction-diffusion (resin disc, δ^r^ = 0.4 mm) domains as derived in Uribe et al. (2011), where *k*_d_ indicates the corresponding dissociation constant of the considered complex pool.

(16) $$\begin{array}{l} \xi =1-\frac{\left(1+\varepsilon K^{\prime }\right)}{\varepsilon K^{\prime }+\frac{\Delta g+\delta^{\text{dbl}}}{\sqrt{\frac{D_{\text{ML}}}{k_{\text{d}}\left(1+\varepsilon K^{\prime }\right)}}}\coth\left(\frac{\Delta g+\delta^{\text{dbl}}}{\sqrt{\frac{D_{\text{ML}}}{k_{\text{d}}\left(1+\varepsilon K^{\prime }\right)}}}\right)+\frac{\Delta g+\delta^{\text{dbl}}}{\sqrt{\frac{D_{\text{ML}}}{k_{\text{d}}}}}\left(1+\varepsilon K^{\prime }\right)\tanh\left(\frac{\delta^{\text{r}}}{\sqrt{\frac{D_{\text{ML}}}{k_{\text{d}}}}}\right)} \end{array}$$

The thicknesses of the diffusion domain (Δ*g* + δ^dbl^) were computed with the filter thickness (0.125 mm), the DBL thickness (0.2 mm) and the corresponding diffusive gel thickness.

**Table 3:** Lability degrees (ξ) of metal complexes obtained in the Mediterranean Sea and in the Belgian coastal Zone (BCZ) (Gao et al., 2019).

**Element**	***c*_**T, M**_**	**Free metal**	**DGT no gel (g0)**	**DGT thin gel (g4)**	**DGT-classic (g8)**	**$\xi_{\text{in}}^{\text{g}0}$**	**$\xi_{\text{org}}^{\text{g}0}$**	**$\xi_{\text{in}}^{\text{g}4}$**	**$\xi_{\text{org}}^{\text{g}4}$**	**$\xi_{\text{in}}^{\text{g}8}$**	**$\xi_{\text{org}}^{\text{g}8}$**
	**nmol L^**−1**^**	**nmol L^**−1**^**	**nmol L^**−1**^**	**nmol L^**−1**^**	**nmol L^**−1**^**						
**MEDITERRANEAN SEA**											
Cd	0.066	0.0029	0.049		0.062	0.694	–			0.89	–
Fe	5.54	5.3 × 10^−13^	0.47		–	–	0.152			–	–
Ni	4.9	0.49	2.95		3.18	0.88	–			0.96	–
Co	0.12	0.019	0.058		0.064	0.81	–			0.94	–
Cu	4.53	0.0044	0.41		0.98	0.70	0.14			0.89	0.36
**BELGIAN COSTAL ZONE**											
Pb	0.473	0.0047	0.0458	0.0644	0.073	0.22	3.8 × 10^−10^	0.40	1.1 × 10^−9^	0.51	2.0 × 10^−9^
Fe	205	1.6 × 10^−9^	37.3	66.6	72.9	–	0.34	–	0.53	–	0.64
Ni	39.5	5.53	5.65	8.17	9.91	0.04	0.04	0.11	0.10	0.18	0.17
Cu	27.7	0.070	3.62	7.61	8.09	0.38	0.14	0.58	0.27	0.68	0.37
Zn	180	10.8	33.6	45.0	50.2	0.96	0.07	0.98	0.16	0.99	0.25

*c_T,M_ is the total dissolved metal concentration; values for Co from Dulaquais et al. (2017) and for the other metals from Ebling and Landing (2015) in the case of the Mediterranean Sea and our data in the case of BCZ. Percentages of each pool (free, inorganic, and organic) are from Stockdale et al. (2011, 2015, 2016). “in” stands for inorganic and “org” for organic. Superscripts: g0 indicates no hydrogel layer, g4 0.4 mm and g8 0.8 mm hydrogel layer*.

It is worth noticing that the lability degree is not entirely an intrinsic parameter of a complex. Even though ξ_*j*_ is a function of the dissociation rate constant and diffusion coefficient (which are, indeed, specific of the complex), it also depends on the geometric characteristics of the sensor (Δ*g*) and the composition of the media (e.g., ligand concentrations). Nevertheless, the presence of concomitant complexes of the same element has usually a low influence on the total accumulation, since the impact of a complex on another one is mutually opposite (ca. 10% or lower), particularly under ligand excess conditions (see Altier et al., 2018).

Using Equations (2) and (13) to (16) with literature data on concentrations (see Gao et al., 2019 for details), the lability degrees and dissociation rates of the metal complex pools in the BCZ were derived. A value of ε_in_ = 1 was taken as well as a literature value of *D*_ML,org_ (Balch and Gueguen, 2015) for the organic pool.

Table 3 shows results for BCZ. As expected, labilities increase when increasing the thickness of the diffusive gel, when other factors are fixed. For a given metal and DGT configuration, the inorganic pool is always more labile than the organic one, as expected. Table 3 allows to suggest that the lability of the metal complexes might in general be higher in open sea (Baeyens et al., 2018) than in the BCZ. This could be related to the presence of strong ligands released from industrial activities in the harbor at the BCZ. The lability degrees of inorganic Cd, Ni, Co, and Cu complexes in the Mediterranean Sea are high (around 90%) for the classic DGT. Something alike happens with inorganic Zn complexes in the BCZ, while inorganic Cu and Pb lability degrees (in the classic DGT) drop to 68 and 51%, respectively. Labilities of organic Cu complexes are equal in the BCZ and in the Mediterranean Sea. Lability degrees of organic Fe complexes are higher in the BCZ than in the Mediterranean Sea.

In high ionic strength solutions, some drawbacks may occur. Perfect sink conditions are not always met, such as for example the DGT accumulation of Mn in seawater or in other solutions with high Ca or Mg concentrations (Altier et al., 2016). In that case, Mn concentration calculations should be carried out with a modified version of Equation (2), taking into account the competition of Mn with Ca and Mg ions for the resin sites, or via specific calibration curves of Mn (same solution composition and same exposure time as for field samples) accumulated on the DGT resin *vs*. the Mn concentration in seawater.

### Speciation Results in Freshwater Ecosystems

**Figure 4:**
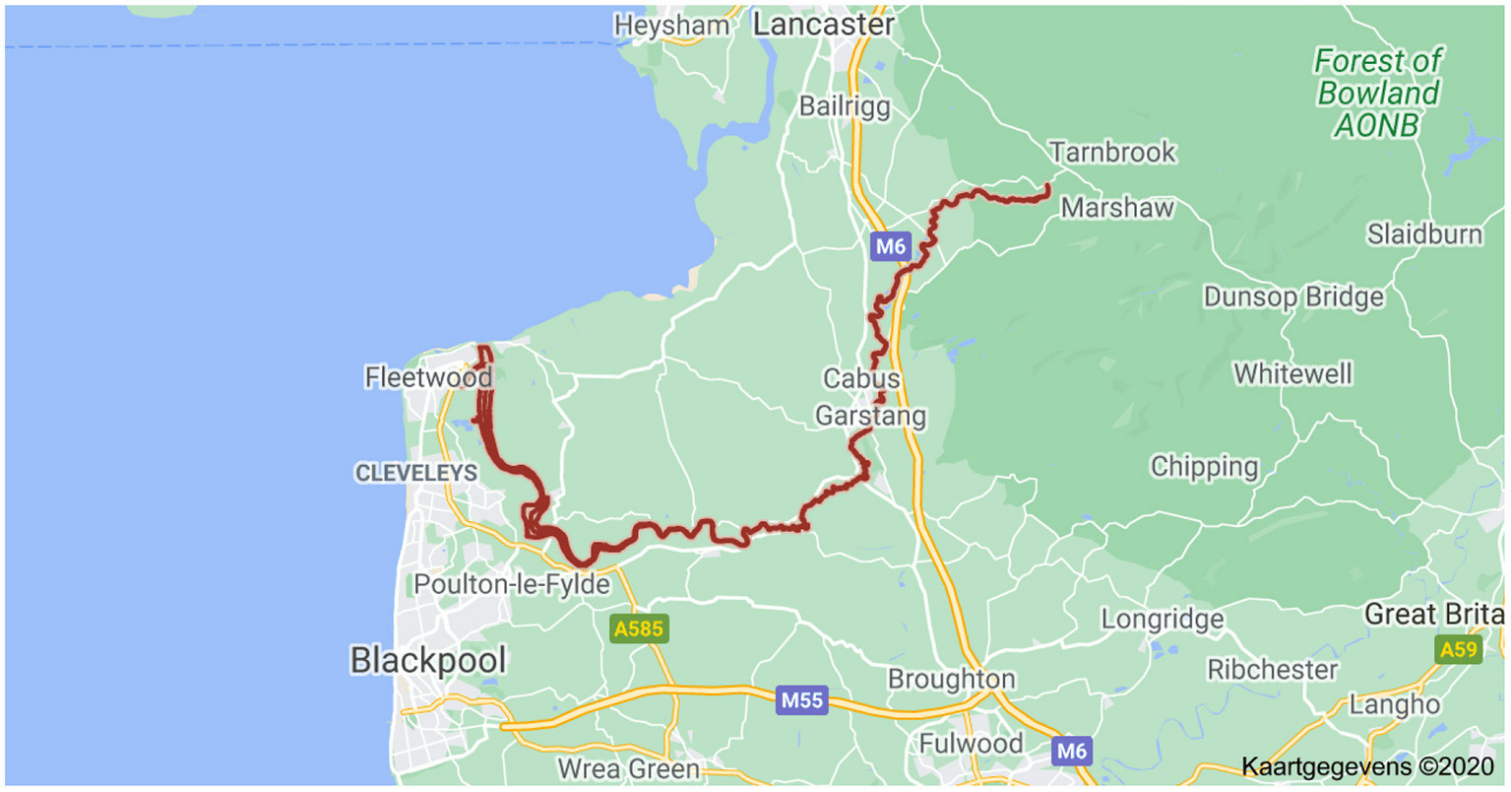
Sampling station at Garstang in River Wyre, Lancaster, UK (Google maps).

Diffusive Gradients in Thin-films was used *in-situ* in the river Wyre (UK, see Figure 4), a pristine freshwater ecosystem with a high DOC level (15 mg/L), to study: (1) the equilibrium distribution of metal ions amongst three pools: simple inorganic complexes (free cation plus complexes with inorganic ligands), organic complexes with fulvic acid (FA), and non-dynamic (inert) metal species (particles, colloids); and (2) the rates of dissociation of the organic complexes. Diffusive Gradients in Thin-films devices were deployed with different diffusion layer thicknesses (0.3, 0.54, 1.34, and 2.14 mm) and with gels of different pore size (either polyacrylamide of normal pore size or restricted gel of small pore size) (Warnken et al., 2008). All samples, including dissolved trace metal samples and DGT eluant samples, were analyzed using a Thermo X7 series. Accuracy and precision were verified by analyzing the National Research Council Canada (NRCC) river water reference material, SLRS-4; the obtained values were within the 95% confidence intervals reported.

The seven spot samples collected during the DGT deployments and used to calculate the mean total dissolved metal concentrations had a RSD between 10% (Fe) and 43% (Mn). However, this is not a precision error, as replicate analyses were within 5%. Rather, it reflects the changes in concentration that are already taken into account in the integrating DGT measurements. The RSD (%) on the DGT measurements (*n* = 10) are between 11% (Ni and Pb) and 17% (Co and Cu). No RSD-values on the classic DGTs were reported, but we found during a cruise in the Southern Ocean (Baeyens et al., 2011) that they are generally lower (maximum of 21%), than the RSD-values on the restricted DGTs (maximum of 57%).

Labile metal concentrations of Mn, Fe, Co, Ni, Cu, Cd, and Pb were calculated assuming inorganic complexes to be fully labile and sharing the same diffusion coefficient as the free metal, while inert complexes did not contribute to the DGT accumulations. These calculations provided the inorganic and organic concentrations and the dissociation rate constant of the organic complexes.

To account for the reduced accumulation found with partially labile complexes, the authors used the approach of an extra diffusion length specific for each element and associated to the kinetic limitation. This length can be written in terms of the dissociation rate constant *k*_d_ and concentrations of real species, as discussed in Warnken et al. (2007, 2008), Mongin et al. (2011), Uribe et al. (2011), Levy et al. (2012), and Puy et al. (2016). This approach can be seen as parallel to the treatment applied in section Speciation Results in Marine Water Ecosystems. Although either ξ or the extra kinetic limitation length depend on the thickness of the diffusive gel used in the DGT device, the number of unknowns is reduced to only *k*_d_ and diffusion coefficients or concentrations because of these theoretical expressions. However, we have to notice that in Warnken et al. (2008), the expression for the extra kinetic limitation length in terms of the dissociation rate constant corresponds to a situation where the complex was not allowed to penetrate into the resin domain, which could be considered a limitation of this treatment. Table 4 shows the percentage distribution of (lumped) inorganic species, *c*_M_ + *c*_ML,in_ (i.e., aggregating the free metal ion concentration with that of the inorganic complexes assumed fully labile and mobile), organic complexes, *c*_ML,org_, and non-dynamic species, *c*_nd_ (calculated as the difference between the total dissolved concentration minus those of the inorganic and organic complexes) in the Wyre river.

**Table 4:** Percentage distribution of lumped inorganic species, (*c*_M_ + *c*_ML,in_) organic Complexes, *c*_ML,org_, and Non-dynamic Species, *c*_nd_ in the Wyre river (Warnken et al., 2008) when taking *D*_ML,org_ as twice of those for isolated fulvic acid complexes.

	**Mn**	**Fe**	**Co**	**Ni**	**Cu**	**Cd**	**Pb**
% (*c*_M_ + *c*_ML,in_)/**TD**	92.2	15.7	30.7	11.3	5.51	27.4	2.01
% *c*_ML,org_/**TD**	9.80	14.7	23.3	65.6	21.8	11.4	0.69
% *c*_nd_ / TD	−1.90	69.6	46.0	23.1	72.6	61.2	97.3

Taking diffusion coefficients of FA-complexes from diffusion cell experiments led to negative non-dynamic concentrations of Mn and Ni. The authors suggested that the mean diffusion coefficients obtained for the isolated FA in the diffusion cell might underestimate the mean diffusion coefficients of complexes that are binding the metals in the river water. So, they performed the final mathematical treatment applying a factor of 2 to all FA-complexes diffusion coefficients (Table 4). The non-dynamic fraction resulted generally in a higher amount than the colloidal fraction measured via ultrafiltration. This may indicate a non-negligible inert metal fraction. More than half of the total filtered Cd was not measured by DGT, consistent with a large colloidal or inert fraction. The authors concluded that Cd is less strongly bound to natural organic material than to extracted fulvic acid. Ni speciation was dominated by DGT-labile organic complexes, in contrast to model predictions made by WHAM6 suggesting that between 40 and 60% of the Ni would be present as inorganic species (Unsworth et al., 2006). Pb behaved quite different than Ni, with a dominant fraction of colloidal species (~97% of total dissolved Pb). The small remaining fraction (~3%) consisted mainly of free Pb and its inorganic complexes. Cu also showed a large inert colloidal fraction (~73%), with a non-negligible fraction bound to humic complexes. The authors could not completely compare with literature data for Fe, Co, and Mn. Fe species were found to be polydispersed (Horowitz et al., 1992) and strongly complexed (Gimpel et al., 2003). The predominant inorganic complexation of Mn agreed with previous results (Gimpel et al., 2003), and the abundances of organically complexed and colloidal Co reported in Table 4 were also consistent with previous work.

These two above-commented cases demonstrated that, in principle, it is possible to use a dynamic technique like DGT to obtain simultaneously information on the distribution of species and their rates of exchange. However, several difficulties such as the uncertainty in the diffusion coefficients of metal complexes, the nature of the organic ligands in the natural aquatic system, the stability constants of the metal–organic ligand complexes, etc., render it difficult to reach straightforward conclusions.

## Mercury Speciation

Mercury, as one of the most toxic elements in the environment, has lately attracted great attention due to its high toxicity and widespread occurrence (Morel et al., 1998). The most important chemical forms are elemental Hg, inorganic Hg, monomethylmercury (MeHg), and dimethylmercury (DMHg), which are all toxic, making mercury probably one of the most hazardous pollutants to aquatic biota (Akagi and Nishimura, 1991; Mason, 1995; Baeyens et al., 2003). It enters the environment from natural and anthropogenic sources, with coal-fired power plants, waste incinerators, and chlor-alkali plants as the most important ones (Turull et al., 2017b). Although mercury is essentially discharged in the inorganic form, it can be converted by sulfate reducing bacteria into methylmercury (MeHg), the most toxic mercury compound in the environment, bio-accumulated in plankton and biomagnified through the aquatic food chain (Mason, 1995; Storelli et al., 2002; Baeyens et al., 2003). The main exposure route of Hg to humans is through the consumption of marine fishery products (Oken et al., 2005). Fish and fish products account for most of the organic mercury in food. It is important to note that fish species can contain a broad range of mercury levels, even in the same aquatic environment.

Bioavailability depends on the chemical lability, but also on passive, active or facilitated cellular interaction with both Hg^2+^ and MeHg, a mechanism which is not yet completely understood (Schaefer and Morel, 2009; Faganeli et al., 2014). Due to the fact that Hg bioavailable species cause ultimately damage to human and ecosystems health, clearly more research into Hg bioavailability is needed (Bradley et al., 2017). Size-fractionating filtration to determine dissolved Hg is often employed to describe the labile fraction that interacts with biota (Faganeli et al., 2003). Similarly, the easily reducible (labile) fraction of Hg^2+^ (referred to as reactive Hg) is used as proxy for photo- and bioreduction, and biomethylation (Horvat et al., 1999). Likewise, experiments of methylation and demethylation potential are based on the assumption that the added non-complexed Hg species is bioavailable (Hines et al., 2006). Moreover, the ratio between MeHg and total mercury (THg) in various aquatic systems is sometimes used to compare the methylation potential, and thus, by extent, the bioavailability of the precursor, although methylation may occur also extracellularly and abiotically (Hines et al., 2006).

Diffusive Gradients in Thin-films has been increasingly used to evaluate lability and, by approximation, bioavailability of and potential exposure to Hg species (Amirbahman et al., 2013; Fernández-Gómez et al., 2015). Hg^2+^ and MeHg in natural waters are often present in femtomolar (fmol L^−1^, pg L^−1^) concentrations, which require ultra clean passive sampling methods and very sensitive, precise analytical procedures. Diffusive Gradients in Thin-films technique is capable of long-term passive accumulation of labile Hg species onto a binding resin, effectively pre-concentrating inorganic Hg and MeHg at such ultralow concentrations in aquatic environments, thus avoiding issues associated with trace metal sampling (Docekalova and Divis, 2005; Clarisse and Hintelmann, 2006; Gao et al., 2014).

The combination of the polyacrylamide diffusive gel and Chelex-100 resin gel, which is commonly used in DGT for the assessment of most trace metals as seen in section Transition Metals, has also been applied for the determination of labile Hg concentrations (mainly inorganic Hg) in the aquatic environment (Cattani et al., 2008), but several drawbacks appeared. The polyacrylamide gel showed a high affinity for mercury and was therefore replaced by an agarose gel (Docekalova and Divis, 2005). Moreover, the adsorption efficiency of the Chelex-100 resin for Hg is also controversial. Docekalova and Divis (2005) compared the Hg pre-concentration by a DGT containing a Chelex-100 resin with one containing a Spheron-Thiol resin (Slovák et al., 1979). They concluded that they were able to assess ionic mercury and labile mercury complexes with both resins, but the Chelex-100 resin had a lower accumulation rate than the Spheron-Thiol one (Gao et al., 2014). Moreover, Cattani et al. (2008) reported that using Chelex-100 as the resin gel in the DGT, only 50–58% of the Hg could be recovered from their solution, while this was 83–97% with the Spheron-Thiol resin. Other thiol-based resins such as mercaptopropyl (Howard and Khdary, 2004; Merritt and Amirbahman, 2007; Clarisse et al., 2009) or dithiocarbamate-based resins (Lansens et al., 1990; Goubert-Renaudin et al., 2007) have been investigated, all with good success, for the pre-concentration of inorganic and organic mercury species at ng L^−1^ level. However, many of them are not or no longer commercially available due to interrupted production; only 3-mercaptopropylsilica (3-MFS) and Purolite S924 are still commercially available (Gao et al., 2011; Bratkic et al., 2019; Reichstädter et al., 2020). However, individual research groups have synthesized new home-made resins for assessment of Hg and other trace metal in aquatic systems (Gao et al., 2011; Turull et al., 2017a; Elias et al., 2020; Reichstädter et al., 2021). Due to the very strong binding between the functional groups of the resin and Hg, the elution procedures for Hg are also different from those for other trace metals (elution procedures can be found in Table 5). It is clearly shown that aqua regia digestion of resin gels produced the most uniform and efficient elution results. Employment of higher HCl concentrations also improve the elution of Hg.

**Table 5:** Extraction procedures for dissolved labile mercury.

**Eluent**	***f*_**e**_**	***T* (°C)**	**Time**	**Analyser**	**References**
10% NaOH	60–75%	N/A	N/A	CV-AAS	Minagawa et al., 1980
30% NaOH	60–75%	N/A	N/A	CV-AAS	Minagawa et al., 1980
2% thiourea in 0.1 M HCl	54%	N/A	24 h	CV-AFS	Ren et al., 2018
1.3 mM thiourea in 0.1 M HCl	25%	N/A	24 h	CV-AFS	Ren et al., 2018
5% thiourea in 0.5% HCl	100%	N/A	N/A	CV-AAS	Minagawa et al., 1980
10% thiourea in 0.1 M HCl	21%	N/A	24 h	CV-AFS	Ren et al., 2018
1 M thiourea	<30%	N/A	N/A	ICP-AES	Pohl and Prusisz, 2004
2 M thiourea in 0.5 M HCl	<30%	N/A	N/A	ICP-AES	Pohl and Prusisz, 2004
1 M HNO_3_	68%	25°C	24 h	ICP-MS	Reichstädter et al., 2020
2 M HNO_3_	16%	N/A	24 h	CV-AFS	Ren et al., 2018
2 M HNO_3_	91%	N/A	24 h	CV-AFS	Colaco et al., 2014
2 M HNO_3_	16%	N/A	24 h	CV-AFS	Ren et al., 2018
3 M HNO_3_	8%	N/A	N/A	ICP-AES	Sook-Young et al., 2001
1 M HCl	5–20%	N/A	24 h	CV-AFS	Noh et al., 2019
2 M HCl	0.5–5.2%	N/A	N/A	ICP-AES	Pohl and Prusisz, 2004
2 M HCl	5–20%	N/A	24 h	CV-AFS	Noh et al., 2019
2 M HCl	17%	N/A	24 h	CV-AFS	Ren et al., 2018
3 M HCl	19%	N/A	24 h	CV-AFS	Ren et al., 2018
5 M HCl + microwave	95%	55°C	15 min	LC-CV-AFS	Pelcová et al., 2015
6 M HCl	100%	N/A	24 h	CV-AFS	Noh et al., 2019
12 M HCl	54%	110°C	5 h	CV-AFS	Fernández-Gómez et al., 2011
7:3 HNO_3_:H_2_SO_4_	92%	110°C	5 h	CV-AFS	Fernández-Gómez et al., 2011
7:3 HNO_3_:H_2_SO_4_	74%	N/A	24 h	CV-AFS	Noh et al., 2016
H_2_SO_4_, conc. w/ 30% H_2_O_2_	92–101%	N/A	N/A	ICP-AES	Pohl and Prusisz, 2004
Aqua regia	96%	N/A	overnight	ICP-MS	Bratkic et al., 2019
Aqua regia	97%	110°C	5 h	CV-AFS	Fernández-Gómez et al., 2011
Aqua regia	88%	N/A	24 h	CV-AFS	Ren et al., 2018
Aqua regia	98%	70°C	24 h	ICP-MS	Reichstädter et al., 2020
Aqua regia	84%	N/A	24 h	CV-AFS	Noh et al., 2016
10% sodium sulfide	60–75%	N/A	N/A	CV-AAS	Minagawa et al., 1980
5% disodium-EDTA	20–30%	N/A	N/A	CV-AAS	Minagawa et al., 1980
5% pentasodium-DTPA	20–30%	N/A	N/A	CV-AAS	Minagawa et al., 1980
10% BrCl	101%	N/A	24 h	CV-AFS	Amirbahman et al., 2013
0.3 M BrCl	90%	N/A	24 h	CV-AFS	Noh et al., 2016

*The extraction efficiency (f_e_), temperature (T), time of extraction, and the analyzing method are detailed*.

Diffusive Gradients in Thin-films determination of mercury is, compared to DGT determination of other trace metals, still under development. Therefore, the validation of the whole methodology is strongly recommended. Considering the instruments used for Hg analysis, it varies from one laboratory to another due to available facilities. Cold Vapor coupled with Atomic Absorption Spectrometry (CV-AAS), ICP-AES, and ICP-MS are commonly used devices in several studies (see Table 5).

Due to the higher toxicity of organic mercury species, especially methylmercury (MeHg), it is important to detail speciation of the Hg compounds in environmental samples. However, in aquatic systems, MeHg occurs at ultra-low concentration levels, which makes the DGT a promising technique for its assessment. Only a few studies have been published on MeHg determination using DGT samplers, which contained polyacrylamide diffusive and Spheron-Thiol resin gel (Liu et al., 2012; Tafurt-Cardona et al., 2015). In their studies, high uncertainties were obtained for water concentration estimates. Diffusion coefficients in the gel appeared to be variable and this might be linked to the employment of polyacrylamide, since it was reported that this gel binds inorganic mercury and MeHg (Docekalova and Divis, 2005; Gao et al., 2014). Moreover, the utilization of thiourea to elute the Hg species from the gel can strongly interfere the ethylation reaction of MeHg (see Table 6), which is a key step for the measurement of MeHg using Cold Vapor-AAS or Headspace-Gas Chromatography-Atomic Fluorescence Spectrometry. Alternatively, agarose was employed as a diffusive gel in the DGT, while detection was carried out with IC-ICP-MS to avoid an ethylation reaction after the thiourea elution. Moreover, a SPE step was used to extract MeHg from thiol-based resin gels (Table 6).

**Table 6:** Extraction procedures for dissolved methylmercury.

**Eluent**	***f*_**e**_**	***T* (°C)**	**Time**	**Analyser**	**References**
2 M HNO_3_	37%	N/A	24 h	CV-AFS	Ren et al., 2018
1 M HCl	97%	N/A	24 h	CV-AFS	Tafurt-Cardona et al., 2015
2 M HCl	40%	N/A	24 h	CV-AFS	Ren et al., 2018
3 M HCl	43%	N/A	24 h	CV-AFS	Ren et al., 2018
18% KBr, 5% H_2_SO_4_, 1 M CuSO_4_, CH_2_Cl_2_	N/A	N/A	1 h	HS-GC-CV-AFS	Bratkic et al., 2019
18% KBr, 5% H_2_SO_4_, 1 M CuSO_4_, CH_2_Cl_2_	100%	N/A	2.5 h	HS-GC-CV-AFS	Gao et al., 2014
Acidic thiourea	N/A	N/A	24 h	IC-ICP-MS/HPLC-ICP-MS	Hong et al., 2011
0.001 mM thiourea, 0.1 M HCl	32%	N/A	24 h	GC-CV-AFS	Noh et al., 2016
0.01 mM thiourea, 0.1 M HCl	33%	N/A	24 h	GC-CV-AFS	Noh et al., 2016
0.1 mM thiourea, 0.1 M HCl	34%	N/A	24 h	GC-CV-AFS	Noh et al., 2016
1 mM thiourea, 0.1 M HCl	88%	N/A	24 h	GC-CV-AFS	Noh et al., 2016
1.3 mM thiourea in 0.1 M HCl	85%	N/A	24 h	CV-AFS	Amirbahman et al., 2013
1.31 mM thiourea, 0.1 M HCl	N/A	N/A	24 h	CV-AFS	Liu et al., 2012
1.31 mM thiourea, 0.1 M HCl	N/A	N/A	24 h	HS-SPME-Py-AFS	Fernández-Gómez et al., 2014
1.31 mM thiourea	91%	N/A	N/A	GC-ICP-MS	Clarisse and Hintelmann, 2006
1.3 mM thiourea, 0.1 M HCl	68%	N/A	24 h	CV-AFS	Ren et al., 2018
1.3 mM thiourea, 0.1 M HCl	N/A	N/A	12 h	ID-GC-ICP-MS	Bretier et al., 2020
10 mM thiourea, 0.1 M HCl	83%	N/A	24 h	GC-CV-AFS	Noh et al., 2016
0.5–50 mM thiourea	< LOD	N/A	24 h	HS-GC-CV-AFS	Gao et al., 2014
100 mM thiourea, 0.1 M HCl	69%	N/A	24 h	GC-CV-AFS	Noh et al., 2016
1000 mM thiourea, 0.1 M HCl	31%	N/A	24 h	GC-CV-AFS	Noh et al., 2016
2% thiourea, 0.1 M HCl	100%	N/A	24 h	CV-AFS	Ren et al., 2018
10% thiourea, 0.1 M HCl	50%	N/A	24 h	CV-AFS	Ren et al., 2018

*The extraction efficiency (f_e_), temperature (T), time of extraction, and the analyzing method are mentioned*.

Mercury speciation using the DGT has been applied in diverse environments including freshwater (Gao et al., 2011, 2014; Hong et al., 2011; Fernández-Gómez et al., 2014; Tafurt-Cardona et al., 2015; Bretier et al., 2020), seawater (Hong et al., 2011), sediments (Amirbahman et al., 2013; Noh et al., 2016; Bratkic et al., 2019), and paddy soil (Liu et al., 2012). Care must be taken for the selection of the diffusion gel and the accurate determination of diffusion coefficients to obtain meaningful results. Due to different selection of diffusive gels, either polyacrylamide or agarose, the diffusion coefficients for different mercury species vary from one study to another. The diffusion coefficient in water and the hydrogels should theoretically decrease as: water > agarose diffusive gel > polyacrylamide diffusive gel > bisacrylamide-crosslinked polyacrylamide (restrictive) diffusive gel, and this due to the decrease of the gel pore size (Davison and Zhang, 2016). The pore size of the polyacrylamide gel is around 10 nm, with a range of 5–20 nm (Zhang and Davison, 1995; Scally et al., 2006) and the one of the agarose is around 40 nm, with a range of 35–47 nm (Zhang and Davison, 1999; Fatin-Rouge et al., 2004). Therefore, it is logic that the diffusion coefficients of mercury species in agarose gel are larger than those obtained in polyacrylamide diffusive gel. Although speciation of bioavailable mercury is technically feasible, careful selection of hydrogel and binding resin for the DGT and appropriate lab processing and analysis should be warranted. Also, biofilm formation with agarose gel is still problematic for long deployment times, which are sometimes necessary due to the very low *in-situ* concentrations of MeHg.

## Saturation/Competition Effects

Diffusive Gradients in Thin-films devices were initially designed to obtain linear accumulations of metals in natural unpolluted waters using deployment times of the order of days. However, too long deployments or deployments in highly contaminated waters can cause saturation effects. These are clearly evidenced by recording the time dependence of the accumulation. Saturation effects lead to downwards deviations of the accumulation indicating that the flux is decreasing with time and the accumulation proceeds out of steady state.

The arising of saturation effects is, then, a phenomenon that depends on the deployment time, the concentrations of the target analyte in the sample, the effective capacity of the resin disc and the affinity of the analyte for the resin sites.

As for the effective capacity, it depends not only on the amount of resin used in the disc, but also on the presence of competing cations. Indeed, natural samples usually consist of mixtures of multiple analytes and ligands. Protons and other concurrent metal cations compete with each other and with the probe metal ion(s) for the binding to the resin sites. The subsequent decrease in the concentration of available (free) resin sites leads to a drop in the effective rate of association between the analyte and the resin (Jimenez-Piedrahita et al., 2017) and to a decline in the rate of metal accumulation in the DGT device. At longer times, the accumulation approaches the effective equilibrium value between the resin and the bulk solution concentration:

(17) $$\begin{array}{l} \frac{c_{\text{MR}}^{\text{eq}}}{{\text{c}}_{\text{T},\text{R}}}=\frac{K_{\text{MR}}c_{\text{M}}}{1+K_{\text{MR}}c_{\text{M}}} \end{array}$$

where *c*_T,R_, $c_{\text{MR}}^{\text{eq}}$, and *K*_MR_ are the total concentration of resin sites, the concentration of sites occupied by M at equilibrium, and the metal-resin binding constant, respectively. The previous equation strictly holds when only the species M is bound to the resin. In a general situation, it should be substituted by a suitable competitive isotherm. In the particular case of competition between M and protons with a stoichiometric exchange ratio of 1:1, the intrinsic value of *K*_MR_ may be replaced with a conditional (effective) value that depends on pH and incorporates the decrease in the number of available sites due to protonation. The competitive effect is, then, interpreted as a lowering of this effective binding constant as pH decreases, which leads to a lower value of $c_{\text{MR}}^{\text{eq}}$ and, consequently, to a more significant curvature of the accumulation plot at low deployment times. Equation (17) is included here as an example of how the effective binding capacity of the resin may be significantly lower than the absolute (intrinsic) binding capacity (i.e., $\frac{c_{\text{MR}}^{\text{eq}}}{c_{\text{T},\text{R}}}<1$) depending on the environmental conditions of the sample.

Moreover, a significant concentration of dissolved ligands with an affinity high enough to compete with resin sites for the binding of the probe ion(s) (Mongin et al., 2013) might also lower the effective strength of the resin, leading again to a curvature of the mass accumulation *vs. t* plot as it approaches the equilibrium value:

(18) $$\begin{array}{l} c_{\text{MR}}^{\text{eq}}=\frac{K_{\text{MR}}c_{\text{ML}}c_{\text{R}}}{K_{\text{ML}}^{\prime }} \end{array}$$

where *c*_R_ is the concentration of free resin sites and $K_{\text{ML}}^{\prime }$ is the effective binding constant of the complex with the high-affinity ligand. The larger the value of $K_{\text{ML}}^{\prime }$, the sooner the accumulation deviates from linearity.

Any of the previous two phenomena leads to a departure from the linear accumulation regime (at shorter times than expected for a significant saturation of the resin) and to an underestimation of the actual species concentration in solution. Mongin et al. (2013) reported some guiding contour plots to help define the range of experimental conditions (pH, $K_{\text{ML}}^{\prime }$) where the linear accumulation regime prevails. As an example, the relative error in the calculation of *c*_DGT_ with the steady-state, perfect-sink (linear) Equation (2) remains below 5% (as compared with an “exact” value computed from numerical simulation results) after 10 h of deployment at pH 7 or higher in solutions containing a strong (but fully labile) complex with $K_{\text{ML}}^{\prime }>3$. For partially labile systems, the range of deployment times where the linear behavior is valid becomes multiplied by 1/ξ_ML_.

## *c*_DGT_ As a Time-weighted Average Concentration

Another issue of practical interest is the elucidation of the relationship between the measured value of *c*_DGT_ and the value of the bulk concentrations averaged over the deployment time. Temporal variations in the concentration of pollutants are frequent in natural waters as a result of the dynamics of natural and anthropogenic cycles. The integrative nature of DGT passive samplers suggests that they can provide time-weighted average (TWA) concentrations of the analytes over the deployment time, in contrast with conventional grab sampling (which yields a snapshot of the sampling site at one particular moment; Allan et al., 2007; Dunn et al., 2007; Huang et al., 2016, 2017). The response of DGT devices to changes in concentration during the deployment time has been studied both theoretically and experimentally through DGT deployments in solutions of different concentrations during controlled time periods (Altier et al., 2019). The results indicate that, if the duration of a pulsed fluctuation is much longer than the characteristic time to reach steady state (*ca*. 10 min in the standard DGT configuration), the transient effects can be neglected. Divergences between *c*_DGT_ and the metal concentration increase as the duration of successive concentration jumps decreases, although the errors may cancel out in the case of regular, periodical fluctuations.

When transient effects are negligible and the deployment takes place under perfect-sink conditions (linear accumulation regime), it can be seen that *c*_DGT_ corresponds to the time-weighted average of the labile concentration in the sample (Altier et al., 2019). This general result, together with the expression of *c*_DGT_ in terms of the concentration of real species, Equation (3), leads to a general equation for the interpretation of *c*_DGT_ in fluctuating systems. For a sample containing a mixture of *h* different ligands:

(19) $$\begin{array}{l} c_{\text{DGT}}=\langle c_{\text{M}}\rangle +\overset{h}{\underset{j=1}{\sum }}\frac{D_{{\text{M}}^{j}\text{L}}}{D_{\text{M}}}\langle \xi_{j}c_{{\text{M}}^{j}\text{L}}\rangle \approx \langle c_{\text{M}}\rangle +\overset{h}{\underset{j=1}{\sum }}\frac{D_{{\text{M}}^{j}\text{L}}}{D_{\text{M}}}\xi_{j}\langle c_{{\text{M}}^{j}\text{L}}\rangle \end{array}$$

where angle brackets are used to denote averages over time. This equation can be seen as applying averages to Equation (3). The approximation in the last r.h.s. of this equation was carried out under the assumption that the dependence of the lability degrees with concentration is negligible (which is reasonable in excess of ligand conditions). In the particular case of only one strong ligand, where the free metal concentration can be neglected, the previous equation simplifies to:

(20) $$\begin{array}{l} c_{\text{DGT}}\approx \frac{D_{\text{ML}}}{D_{\text{M}}}\xi_{\text{ML}}\langle c_{\text{ML}}\rangle \end{array}$$

Equation (20) allows to correct *c*_DGT_ in order to obtain a better estimation of the metal concentration in the sample when the diffusion coefficient and the lability degree of the complex are known, as has been shown for the case of Ni in solutions with nitrilotriacetic acid (Altier et al., 2019).

## Conclusions

Diffusive Gradients in Thin-films has evolved, since the pioneering work of Davison and Zhang (1994), to become a mature technique for *in-situ* measurement of the availability of metal cations, anions and, more recently, organic compounds in natural waters, soils, and sediments. In addition to the simplicity of this technique, an attractive characteristic is its ability to determine labile concentrations from the knowledge of the diffusion coefficient of the target analytes and the thickness of the diffusive domain (DBL+filter+hydrogel layer). The diffusive domain regulates the metal flux arriving to the resin gel, so that the association rate constants between the target analyte and the resin sites do not influence the accumulation.

Deployment of DGTs in freshwater and seawater allowed the characterization of inorganic and organic trace metal complexes. Labile fractions varied for each element according to the conditions in the aquatic environment (load and nature of organic ligands, redox and pH conditions, ionic strength, etc.). Using DGT devices with different thicknesses (together with complementary techniques) opens the way to an even more detailed characterization of the trace metal availability in natural media. Mercury speciation has benefited from new binding agents and improved elution and detection of the accumulated amount, although more work is still needed.

Amongst further developments to improve the speciation of inorganic species in the aquatic environment, we can highlight: (1) lowering the detection limits for open ocean, by lowering the blanks or by increasing the amount accumulated on the resin e.g., by using ultra-thin diffusive domains; (2) better knowledge of major groups of organic ligands with performant organic analytical instruments; (3) more efficient binding materials for the different analytes; and (4) combination of complementary techniques to build up an integrated description of equilibrium and dynamic speciation.

## Author Contributions

JG contributed to modeling, validation, investigation, and writing. YG contributed to supervision, writing, data analysis, reviewing, and editing. JP contributed to supervision and writing. ML contributed to analysis, data analysis, and editing. CR-C contributed to modeling, software, and editing. CZ contributed to data analysis and visualization. WB contributed to supervision, writing, and experimental design. All authors contributed to the article and approved the submitted version.

## Conflict of Interest

The authors declare that the research was conducted in the absence of any commercial or financial relationships that could be construed as a potential conflict of interest.
